# The Binomial Parasite-Host Immunity in the Healing Process and in Reactivation of Human Tegumentary Leishmaniasis

**DOI:** 10.3389/fmicb.2018.01308

**Published:** 2018-06-19

**Authors:** Fatima Conceição-Silva, Jessica Leite-Silva, Fernanda N. Morgado

**Affiliations:** ^1^Laboratory of Immunoparasitology, Oswaldo Cruz Institute (Fiocruz), Rio de Janeiro, Brazil; ^2^Laboratory of Leishmaniasis Research, Oswaldo Cruz Institute (Fiocruz), Rio de Janeiro, Brazil

**Keywords:** tegumentary leishmaniasis, healing process, lesion reactivation, patients, leishmaniasis, *Leishmania* parasites, co-morbidities, immunosuppression

## Abstract

Leishmaniasis is a vector-borne infectious disease caused by different species of protozoa from the *Leishmania* genus. Classically, the disease can be classified into two main clinical forms: Visceral (VL) and Tegumentary (TL) leishmaniasis. TL is a skin/mucosal granulomatous disease that manifests mainly as cutaneous localized or disseminated ulcers, papules diffusely distributed, mucosal lesions or atypical lesions. Once the etiology of the infection is confirmed, treatment can take place, and different drugs can be administered. It has already been shown that, even when the scar is clinically evident, inflammation is still present in the native tissue, and the decrease of the inflammatory process occurs slowly during the 1st years after clinical healing. The maintenance of residual parasites in the scar tissue is also well documented. Therefore, it is no longer a surprise that, under some circumstances, therapeutic failure and/or lesion reactivation occurs. All over the years, an impressive amount of data on relapses, treatment resistance and lesion reactivation after healing has been collected, and several factors have been pointed out as having a role in the process. Different factors such as *Leishmania* species, parasite variability, *Leishmania* RNA virus 1, parasite load, parasite persistence, age, nutritional status, gender, co-morbidities, co-infection, pregnancy, immunosuppression, lesion duration, number and localization of lesions, drug metabolism, irregular treatment and individual host cellular immune response were described and discussed in the present review. Unfortunately, despite this amount of information, a conclusive understanding remains under construction. In addition, multifactorial influence cannot be discarded. In this context, knowing why leishmaniasis has been difficult to treat and control can help the development of new approaches, such as drugs and immunotherapy in order to improve healing maintenance. In this sense, we would like to highlight some of the findings that may influence the course of *Leishmania* infection and the therapeutic response, with an emphasis on TL.

## The Problem

Leishmaniasis is a vector-borne infectious disease caused by different species of the *Leishmania* genus. Classically, the disease is classified into two main clinical forms: Visceral (VL) and Tegumentary (TL) leishmaniasis. Although VL is more severe, TL is much more spread all over the world. According to the World Health Organization (WHO), cases of leishmaniasis can be found in 98 countries with approximately 0.7–1 million cases of TL and 200–400 thousand cases of VL per year (Alvar et al., 2012; World Health Organization [WHO], 2017a).

**FIGURE 1:**
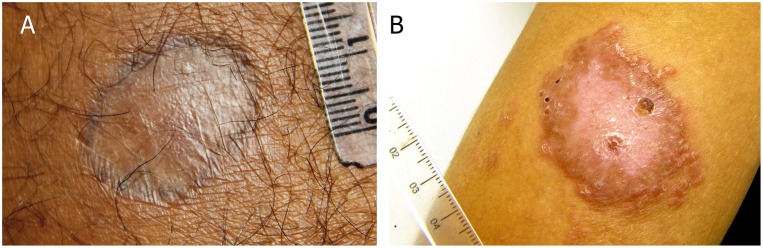
Tegumentary leishmaniasis caused by *Leishmania braziliensis*. **(A)** Atrophic scar post a successful treatment; **(B)** lesion reactivation post treatment. Photos kindly provided by Dr. M. R. Lyra and LaPClin Vigileish - National Institute of Infectology Evandro Chagas -INI- Fiocruz.

Tegumentary leishmaniasis is a skin/mucosal granulomatous disease that manifests as cutaneous localized or disseminated ulcers, papules diffusely distributed, mucosal lesions or atypical lesions (**Table 1**) (Marzochi and Marzochi, 1994; Azeredo-Coutinho et al., 2007; Kevric et al., 2015; Reis et al., 2016; Brazil, 2017; Carvalho et al., 2017). Marzochi and Marzochi (1994) suggested a clinical approach based on clinical and evolutionary aspects as follow: (1) Localized cutaneous leishmaniasis (LCL), most commonly manifestation of tegumentary leishmaniasis, it is characterized by single or multiple ulcers with varying measures of mm to cm. In most cases, it is located in exposed areas and at the site of inoculation of the parasite. The typical ulcer is painless, of varied size, has delimited and raised edges of granular bottom. These lesions may regress spontaneously after some time (Costa et al., 1987; Oliveira-Ribeiro et al., 2017) or by use of specific therapies. The scar, in general, has a smooth, thin, shiny and hypopigmented appearance (**Figure 1A**) (Costa et al., 1987; Schubach et al., 1998). Cases of reactivation of the lesions can be seen in some patients considered clinically cured (**Figure 1B**). (2) Disseminated Cutaneous Leishmaniasis (DiL), it is considered one of the rare forms of the disease, characterized mainly by the large number of lesions found by the body ranging from ten to even hundreds (Machado et al., 2011). It initially presents an ulcer similar to the localized cutaneous form and evolves with the appearance of several polymorphic papules, that may have acneiform, nodular, ulcerated, verrucous and plaque appearance. (3) Diffuse Cutaneous Leishmaniasis (DCL), it is a rare form of ATL with multiple non-ulcerated nodular lesions, usually presenting a verrucous, plaque and/or vegetative aspect reaching large body regions. Patients affected by the diffuse form have no cellular immune response to *Leishmania* spp. antigens (Convit et al., 1972), with a Montenegro skin test (MST) negative and a poor response to therapies. (4) Mucosal Leishmaniasis (ML), it is considered serious and usually appears years after the skin lesion, affecting mucous membranes of the nose, mouth, pharynx, and larynx, causing irreversible destructive lesions that can lead to respiratory complications and malnutrition. It is estimated that 3–5% of cases of cutaneous form develop mucosal lesion (Marzochi and Marzochi, 1994; Azeredo-Coutinho and Mendonça, 2014; Brazil, 2017).

**Table 1:** Clinical characteristics of lesions and the *Leishmania* species involved in main clinical forms of TL.

Geographic distribution	Main species	TL clinical forms	Lesion description
New World	*L. (V.) braziliensis L. (L.) amazonensis L. (V.) guyanensis L. (L.) mexicana L. (V.) panamensis L. (V.) lainsoni*	Localized cutaneous leishmaniasis (LCL)	Single or multiple ulcerated lesions located in a single region of the body. The typical ulcer is painless, of varied size, well delimited with raised infiltrated edges and granular bottom, and usually with good response to treatment.
Old World	*L. (L.) major L. (L.) tropica L. (L.) aethiopica*		
New World	*L. (V.) braziliensis L. (L.) amazonenses*	Disseminated leishmaniasis (DiL)	Numerous lesions (≥10), most of them small and ulcerated, distant from the site of the phlebotomine bite. Good/poor response to treatment.
New World	*L. (L.) amazonesis L. (L.) mexicana*	Diffuse cutaneous leishmaniasis (DCL)	Rare form of ATL with multiple non-ulcerated infiltrated or nodular lesions reaching large body regions, usually presenting a plaque, verrucous and/or vegetative aspect.
Old World	*L. (L.) aethiopica*		
New World	*L. (V.) braziliensis L. (L.) amazonensis L. (V.) panamensis*	Leishmaniasis recidiva cútis (LRC)	Repeated reactivation of lesions around or within the scar of the classic cutaneous form of leishmaniasis.
Old World	*L. (L.) tropica*		
New World	*L. (V.) braziliensis L. (L.) amazonensis L. (V.) guyanensis L. (V.) panamensis*	Mucocutaneous leishmaniasis (ML)	Affects mucous membranes of the nose, mouth, pharynx and/or larynx, causing destructive lesions (mainly ulcer with infiltration) that can lead to respiratory complications and malnutrition. Prolonged treatment is usually effective.
Old World	*L. (L.) major*	Late ML	Mucous membranes lesions occur months or years after cutaneous lesions.
		Concurrent ML	Occurrence of simultaneous cutaneous and mucosal lesions.
		Contiguous ML	Mucosal lesions developed by contiguity due to the presence of periorificial cutaneous lesions on the face.
		ML of undetermined origin	Mucous membranes lesions without any active cutaneous lesion or compatible scar or history of previous leishmaniasis cutaneous lesion.


Other less common clinical presentations are described. As such, we can cite the a so-called leishmaniasis recidiva cutis (LRC), which consists of repeated reactivation of lesions around or within the scar of the classic cutaneous form of leishmaniasis (Bittencourt et al., 1993; Oliveira-Neto et al., 1998). The genesis of these different clinical presentations, ranging from single cutaneous lesions with self-limited evolution and tendency to spontaneous healing, to widespread forms that often evolve with difficult treatment and control, has been related to different factors such as parasite species, patient nutrition and health status, number and localization of lesions, as well as an individual host cellular immune response (Silveira et al., 2004; Brazil, 2017). For example, some authors have shown that American tegumentary leishmaniasis (ATL) lesions located in the lower limbs need more time to heal (Oliveira-Neto et al., 1996; Schubach et al., 2005; Oliveira-Ribeiro et al., 2017). It has also been described that early treatment of ATL does not prevent ulcer development (Machado et al., 2002; Unger et al., 2009) and linked to higher rates of therapeutic failure (Unger et al., 2009). In this context, it has recently been verified that short evolution time and Montenegro skin test (MST) with low positivity were associated with cases of therapeutic failure (Antonio et al., 2014).

Once the etiology of the infection is confirmed, treatment can take place, and different drugs can be administered. In Brazil, the most common treatment is an intramuscular or intravenous injection of pentavalent antimonials (Brazil, 2017). In other world regions, amphotericin B, among other possibilities, is also designed to treat leishmaniasis (Tuon et al., 2008; PAHO, 2013; World Health Organization [WHO], 2017a). However, in addition to the well documented potential harmful toxicity of all drugs already described, increased information about resistance to the first and second choice drugs has highlighted the need for new drugs to treat leishmaniasis. But, until now it has not been possible to accomplish a new treatment with less toxicity and at least the same efficacy of the old available drugs. It is important to note that, in parallel to treatment failure, the literature has also evidenced the possibility of an early spontaneous healing without treatment (Oliveira-Ribeiro et al., 2017; Costa et al., 2018). In this context, understanding why leishmaniasis is becoming difficult to treat and control can help to develop new approaches to improve the healing of this infection. Unfortunately, despite an impressive amount of information, a conclusive understanding is still under construction. Our objective is to introduce and discuss some of the information already published regarding the factors that could influence the treatment response in humans with TL. **Table 2** summarizes some findings regarding treatment response.

**Table 2:** Summary of the aspects involved in human TL treatment.

Findings about treatment	Reference^∗^
Cases of resistance to meglumine antimoniate have been increasing over the years	Santos et al., 2002; Rodrigues et al., 2006; Rojas et al., 2006; Tuon et al., 2008; Oliveira et al., 2011; Lindoso et al., 2012; Schubach and Conceição-Silva, 2014; Ponte-Sucre et al., 2017; Vanaerschot et al., 2018
The parasite species, nutrition and health status of the patient, number and location of the lesions, and the cellular immune response of the host can influence the response to treatment	Oliveira-Neto et al., 1996, Oliveira et al., 2013; Faria et al., 2005; Machado-Coelho et al., 2005; Schubach et al., 2005; Morgado et al., 2008, 2015; Chiheb et al., 2012; Ibrahim et al., 2013; Cunha et al., 2015; Azeredo-Coutinho et al., 2016; García-Bustos et al., 2016; Oliveira-Ribeiro et al., 2017; Brazil, 2017; World Health Organization [WHO], 2017b
The early treatment of ATL does not prevent ulcer development	Machado et al., 2002; Unger et al., 2009
A short evolution time and Montenegro skin test (MST) with low positivity were associated with cases of therapeutic failure	Convit et al., 1972; Antonio et al., 2014
The literature has also evidenced the possibility of an early spontaneous healing without treatment	Costa et al., 1987, 2018; Oliveira-Ribeiro et al., 2017
In cases of contraindication or resistance to pentavalent antimony, other drugs such as amphotericin B and pentamidine may be used. In some regions and species of *Leishmania*, these drugs are recommended as first choice because they are more effective than antimonials.	Ameen, 2010; Oliveira et al., 2011; Schubach and Conceição-Silva, 2014; Mosimann et al., 2018


*^∗^The literature on aspects of TL treatment is vast and other articles can be found. Here are just a few.*

## The Healing Process in TL

The presence of a balanced immune response mainly produced by cellular immunity is pointed out as necessary to control parasites and promote wound healing.

Due to difficulties in obtaining tissue smears, many studies have been focused on analyzing the peripheral blood immune response. It is important to note that relevant information has been described correlating the results of the evolution and severity of the lesions, as well as in the therapeutic response. Despite this, in the last years various results were obtained by the evaluation of tissue damages and the organization and activity of the inflammatory process, which brought valuable information. Nylén and Eidsmo (2012) published a review addressing the different organizations of the inflammatory reaction in the different types of TL lesions. Other reviews have previously been published, bringing valuable information to better understand the immune response in TL (Conceição-Silva et al., 2010; Oliveira et al., 2014; Scott and Novais, 2016; Rossi and Fasel, 2018; among others). The involvement of the *in situ* inflammatory response in the formation and maintenance of the lesions has previously been demonstrated in the murine model. In this context, Belkaid et al. (2000) demonstrated in the murine infection that the formation of the ulcer is almost due to the inflammatory process, and not necessarily to the increase in parasite number, since the ulceration arises when the inflammatory process begins to settle and at the same point the parasitic load tends to decrease. In addition, in patients, the balance between types 1 and 2 responses has been identified as a determinant in the evolution of TL to self-limited or severe forms (Awasthi et al., 2004; Gollob et al., 2008).

**FIGURE 2:**
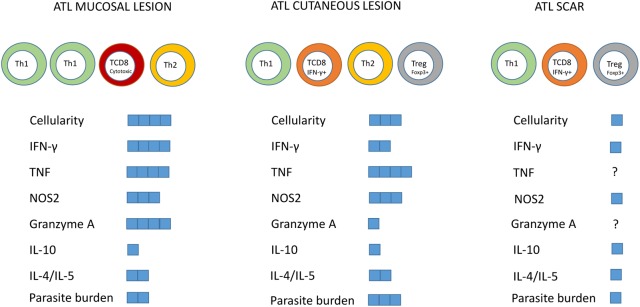
Summary of the main immunological features described in mucosal lesions, localized cutaneous leishmaniasis and skin scars of tegumentary leishmaniasis. Predominant cell subtypes were indicated for each clinical form. The blue squares represent the intensity of the parameters indicated in the figure.

The typical immune response detected in localized cutaneous leishmaniasis patients (LCL) is characterized by the production of proinflammatory cytokines, predominantly Th1 cytokines, such as INF-γ, interleukin (IL) -2 and TNF (Da-Cruz et al., 1996; Gaafar et al., 1999; Coutinho et al., 2002; Gomes-Silva et al., 2007; Kima and Soong, 2013). However, the regulatory response of type 2 cytokines is also presented in the LCL form, by the production of IL-4, IL-5, IL-13, and TGF-β. It is known that an exaggerated Th1 response can lead to tissue damage and has been associated with the immunopathogenesis of mucosal lesions (Ribeiro-de-Jesus et al., 1998; Bacellar et al., 2002; Faria et al., 2005; Palmeiro et al., 2012). Thus, type 1 balanced response with production of cytokines such as IFN-γ, IL-12, and TNF is indispensable for the control of *Leishmania* spp. infection (Nylén and Eidsmo, 2012; Souza et al., 2012; Scott and Novais, 2016). A summary of the main immunological features already described in the tegumentary leishmaniasis is represented in **Figure 2**.

Concerning the tissue microenvironment, it has also been shown that ATL lesions are characterized by a chronic granulomatous inflammatory reaction, with intense lymphoplasmacytic infiltration (Quintella et al., 2012). Lymphocytes, macrophages and neutrophils predominate in the lesions of typical LCL, defined as those with the presence of ulcers with infiltrated borders and granular bottoms (Schubach et al., 2005). Morgado et al. (2008) verified that the inflammatory process was similar in lesions with both, shorter and longer time of evolution, including neutrophils in all patients studied. However, the dynamics of the cellular infiltrate was modified and in the lesions with more than 6 months of evolution it was possible to identify a gradual decrease of CD8^+^ T cells and increase of CD4^+^ T cells associated with the beginning of the healing process and tendency to focal inflammation permeated by areas of fibrosis. These changes could be linked to decreased parasite load (Morgado et al., 2008) and local control of immunological effectors by the accumulation of Treg cells (Campanelli et al., 2006). In this sense, the accumulation of Treg cells, IL-10 and TGF-β in late lesions was also observed (Hejazi et al., 2012; Hoseini et al., 2014). On the other hand, the imbalanced response can both decrease and exacerbate type 1 response that can elicit tissue damage making difficult to control TL. In this context, Maspi et al. (2016) suggested that an immune response with predominance of Type 2 cytokines (IL-4, IL-5) and IL-10 may decrease the expression of the nitric oxide synthase-2 (NOS2) enzyme resulting in insufficient macrophage stimulation to its parasiticide stage, aiding parasitic proliferation and leading to an increase in disease severity as well as to a difficult treatment, as observed in diffuse cutaneous leishmaniasis. Mucosal lesions have been proposed to have an exacerbated and imbalanced cellular immune response with significant increase in lymphocytes, macrophages, Th1 cytokines such as IFN-γ as well as NO (Gomes-Silva et al., 2007; Palmeiro et al., 2012). When compared to cutaneous leishmaniasis, mucosal lesions presented similar amounts of IL-10, NOS2, and TNF, and higher IFN-γ and granzyme A expression accompanied by reduced IL-10 receptor expression (Faria et al., 2005). This uncontrolled response could be responsible for the significant tissue damage, explaining the appearance of extensive, destructive, and difficult-to-treat lesions.

The detailing of the immune response involved in the formation of TL lesions has been the subject of numerous revisions, but some details are important for understanding the mechanism of cure or therapeutic failure. In this context, other cells participate in the inflammatory process and may influence the progression of TL lesions and some have been implicated as involved in both, the control and the pathogenesis of the infection. CD8^+^ T cells are considered important components of the inflammatory infiltrate present in the lesions (Faria et al., 2009; Santos et al., 2013). However, its function has been discussed and recently it has been demonstrated that according to the functional profile, CD8^+^ T cells could play a beneficial (increasing the supply of IFN-γ) or harmful (by the predominance of cytotoxic activity) role (Novais and Scott, 2015; Novais et al., 2018). Neutrophils have also been involved in the decrease in parasite load or in the amplification of macrophage infection (Tacchini-Cottier et al., 2000; John and Hunter, 2008; Conceição et al., 2016). In this context, our group identified the presence of neutrophils in lesions with different time of evolution (Morgado et al., 2008). It was also verified that *Leishmania* spp. induces neutrophil extracellular trap (NET) formation in a parasite load dependence and the leishmanicidal activity of NET was verified *in vitro* (Guimarães-Costa et al., 2009). NET formation was also identified in active lesions of ATL and the presence of two predominant NET sizes related to parasite load was demonstrated (Morgado et al., 2015). Other studies have shown the influence of different cytokines such as IL-17 and enzymes such as arginase in the pathogenesis of TL (Soong et al., 2012; Rath et al., 2014; Banerjee et al., 2016, among others). Although in part these immunological mechanisms together can reduce the local parasite load, *Leishmania* is able to escape from immune effectors then persisting in the lesion site (Makala et al., 2011; Guimarães-Costa et al., 2014). For example, *Leishmania* spp. can escape *in vitro* from the NET-mediated killing through 3′-nucleotidase/nuclease activity (Guimarães-Costa et al., 2014). In fact, intact amastigotes surrounded by NETs were previously observed in active lesions from ATL patients (Morgado et al., 2015).

Skin inflammation also plays an important role during the healing of ATL and parasite antigens in ATL scars have already been reported (Schubach et al., 1998; Mendonça et al., 2004; Morgado et al., 2010). *In situ* evaluation of LCL scars demonstrated that after 1 year from healing, the scars presented inflammatory nests surrounded by scar tissue as well as close to vessels and cutaneous glands. Inflammatory areas presented similar organization than that observed in active lesions from the same patient, including number and distribution of lymphocytes and macrophages, despite a reduction in the inflammatory areas. When scars were examined after 3 year evolution, it was still possible to verify that the inflammatory foci were still present and showing signs of inflammatory activity, including the detection of parasites, but presenting lower cellularity as compared with 1 year scars (Morgado et al., 2010). These results pointed out that the cellular composition of the skin inflammatory reaction changes steadily even after wound healing and, along with the presence of parasites suggests a dynamic balance between parasite multiplication and immune response that could be disrupted in some situations. Thus, individuals with persistent parasites may present disease recurrences (Saravia et al., 1990). In addition, the parasitic persistence may contribute to the continuous immune system stimulation, maintaining a pool of *Leishmania* -specific effector cells which can be detected in peripheral blood of healed patients (Gaafar et al., 1999; Lakhal-Naouar et al., 2015), inducing a protective action (Pereira-Carvalho et al., 2013). The implications of parasite persistence on TL scars become important for the explanation of cases of reactivation of infection, years after clinical cure and will be discussed later in this review.

In the LCL, the cure criterion is clinical and can be defined as healing with complete re-epithelialization, disappearance of crusts, flattening of the borders of the lesions and absence of new lesions (Lindoso et al., 2012; PAHO, 2013), as well as erythema reduction within 3 months of the therapeutic regimen. The healing of the mucosal lesion must be confirmed by otorhinolaryngological examination, until 6 months after the end of the treatment (Gontijo and Carvalho, 2003). In both situations, it is important to monitor the patient at least up to 12 months after clinical cure for early detection of signs of lesion reactivation. Such signs are the absence of complete epithelization until 90 days after the first course of treatment, as well as the worsening of clinical condition and/or the presence of additional lesions. In these cases, the Brazilian Health Ministry recommends a second treatment regimen (Brazil, 2017). It is important to emphasize that, during monitoring, recurrence, therapeutic failure and disease reactivation can be observed. Thus, some authors believe that confirmation of clinical cure is not always satisfactory, due to the occurrence of numerous reports of recurrence even after therapy and total wound healing. It would be interesting to be able to standardize healing criteria not only in relation to clinical parameters, but also parasitological and therapeutic.

## Why Patients Are Not Always Healed?

All over the years, an impressive amount of information has been collected. As consequence, several factors have been implicated as having a role in the process of treatment failure and/or disease reactivation even several years after the clinical cure and it is not possible to discard a multifactorial influence in the response to the specific treatment. Different factors such as *Leishmania* species (Handler et al., 2015), genetic background, age, weight, co-morbidities, lesion duration, number and localization of lesions, drug metabolism, irregular treatment, among others were described (Vera et al., 2001; Rojas et al., 2006; Unger et al., 2009). In this sense, we would like to highlight some of the findings that may alter the course of *Leishmania* infection and modify the therapeutic response in leishmaniasis, with an emphasis on TL.

## Parasite Point of View: Do Parasites Influence the Therapeutic Response?

### Parasite Variability

The *Leishmania* sp. protozoa is considered one of the determining factors of the clinical manifestations (Handler et al., 2015). For example, in the New World the infection by *Leishmania braziliensis* produces localized ulcers whereas the infection by *L. guyanensis* can produces multiple lesions. *L. naiffi*, in general produces benign and auto-resolute lesions that heal spontaneously. Mucosal manifestations occur in patients infected by *L. braziliensis* and *L. guyanensis*, whereas diffuse leishmaniasis occurs in patients infected by *L. amazonensis* (Brazil, 2017). Co-infections with different *Leishmania* species have been described in patients from Manaus, Brazil (Camara-Coelho et al., 2011). However, the impact of co-infections by different *Leishmania* species still needs to be elucidated.

Molecular characterization of *Leishmania* isolates has been vastly carried out by four classical markers: the rRNA internal transcribed spacer 1 (ITS-1), kDNA minicircles, the heat shock protein 70 (HSP70) and the mitochondrial cytochrome b (Cyt-b) through techniques based on the digestion by restriction endonuclease enzymes and sequencing of amplified gene followed by phylogenetic analysis (Buitrago et al., 2011; Cruz et al., 2013; Koltas et al., 2016; Morgado et al., 2016; Bilbao-Ramos et al., 2017). These studies have shown the existence of different genotypes and their relation with clinical manifestations and susceptibility to leishmanicidal drugs. As consequence, disease control could be severely compromised by the intrinsic variability of the circulating species that may limit the efficacy of the treatment while favoring the emergence of resistance. For example, in a study of patients from Bolivia, *L. braziliensis*, *L. lainsoni* and a local variant of *L. mexicana* were isolated and showed differences in the susceptibility to drugs such as Fungizone^®^, Glucantime^®^ and Miltefosine (Bilbao-Ramos et al., 2017). *L. braziliensis* and *L. mexicana* showed moderate sensitivity to Glucantime^®^, whereas *L. lainsoni* was not sensitive to Glucantime^®^.

The different zimodemes detected in *Leishmania* (*Viannia*) isolates from Colombia was associated with clinical and epidemiological characteristics (Saravia et al., 1998). In another study using the randomly amplified polymorphic DNA technique, polymorphisms were detected in isolates from Brazilian patients (Schriefer et al., 2004). All forms of ATL presented a statistically significant difference in their frequencies among the clades, suggesting that *L. braziliensis* genotypes may be accompanied by specific disease manifestation after infection.

Marlow et al. (2014) evaluated 86 strains of *L. braziliensis* from Brazil and identified three populations named POP1, POP2, and POP3 through multilocus sequence analysis (MLSA). The authors observed a significant association between the genetic cluster and the clinical forms of patients. Most strains isolated from patients presenting mucocutaneous form belonged to POP3. Furthermore, 28.6% of strains belonging to POP3 were isolated from patients with mucocutaneous clinical form, whereas 3.1% belonging to POP1 and 5.9% belonging to POP2 were isolated from mucocutaneous patients (Marlow et al., 2014).

### *Leishmania* RNA Virus 1 (LRV1)

Still looking from the parasite side, recently, *Leishmania* RNA virus 1 (LRV1) was identified in isolates from South America and has been associated with the exacerbation of clinical presentations, mucosal lesion emergence and relapses (Cantanhêde et al., 2015; Bourreau et al., 2016; Tirera et al., 2017). LRV was associated to a variety of *Leishmania* species isolated from human patients such as *L. guyanensis*, *L. braziliensis*, *L. aethiopica*, *L. major* (Zangger et al., 2014; Cantanhêde et al., 2015; Adaui et al., 2016; Bourreau et al., 2016; Parmentier et al., 2016; Tirera et al., 2017). The immunological mechanisms behind the worsening of the disease are not fully understood but authors have pointed out the role of endogenous viruses like LRV1 in inducing the expression of type 1 interferon and down-regulation of IFN-γ receptors by macrophages (Rossi and Fasel, 2018). The increase of type 1 interferon in mice leads to an increase in pathogenicity. Associated to the reduction of IFN-γ signal, this profile is not able to fully control the parasite load. Some evidence showed the recognition of LRV1 by TLR3 receptor (Macedo et al., 2016) and consequent upregulation of miR-155 and the promotion of parasite persistence mediated by macrophage survival through akt activation (Eren et al., 2016). The blockade of akt or miR-155 deficiency showed a drastic decrease in LRV1-induced disease severity (Eren et al., 2016).

The presence of LRV1 was also investigated in patients infected with *L. guyanensis* (Bourreau et al., 2016). The authors showed significant prediction of treatment failure in positive cases. However, the presence of *Leishmania* RNA virus 1 infection cannot be considered as exclusively factor inducing severity of tegumentary leishmaniasis in Brazil as observed by Pereira et al. (2013). The authors studied patients from Rio de Janeiro-Brazil, where no LRV1 was detected despite the presence of mucosal patients suggesting that other factors may influence the exacerbation of the disease and mucosal development (Pereira et al., 2013). In this context, the inflammatory response generated by exogenous viruses in mice co-infected with *Leishmania* parasites was demonstrated (Rossi and Fasel, 2018). Furthermore, differences in proteome, metabolome and virulence of isolates from cutaneous and mucosal sites in the same patients were previously observed (Alves-Ferreira et al., 2015). Differences in immune response from nasal and oral lesions were also observed (Palmeiro et al., 2012) Altogether these data suggest the role of particularities from different tissue compartments on the metabolic/physiological pressure on the selection of *Leishmania* clones reflecting in differential clinical manifestations. On the other hand, in areas where *Leishmania* parasites are considered quite homogeneous like Rio de Janeiro, Brazil, different clinical presentations and different degrees of treatment response can be observed, also pointing out that other factors should be involved to determine the evolution of TL (Baptista et al., 2009).

### Parasite Load

Another point to consider is the influence of parasite load on infection control and consequently on the response to treatment. In the murine model it has been described that, depending on the parasite concentration used for the experimental infection, the immune response can be modified, producing self-limited or severe lesions (Bretscher et al., 1992; Hosken et al., 1995; Menon and Bretscher, 1996). Courret et al. (2003) compared intradermic inoculations with different numbers of *L. major* promastigotes varying from 10 to 1000 metacyclic promastigotes and observed that different from mice infected with 10 parasites, inoculations with 100 or 1000 parasites led to progressive lesions in most of infected animals, as well as the resistance to reinfection. In hamster model infected with *L. braziliensis* different concentrations of initial inocula and evaluated during 105 days post infection showed that the lesion emergence occurred early in animals infected with higher inocula concentration (Ribeiro-Romão et al., 2014). However, at the end of the experiment, IFN-γ expression and parasite load were similar between groups, suggesting that immunomodulation takes place early during experimental infection (Ribeiro-Romão et al., 2014).

During the sand fly blood feeding, a mix of promastigotes and saliva is inoculated in the dermis. Some compounds such as promastigote secretory gel (PSG), sand fly saliva, among others, may lead to a better establishment of infection, favoring the parasite survival at the beginning of infection (Almeida et al., 2003; Gomes and Oliveira, 2012; Courtenay et al., 2017). Furthermore, the modulation of host immune response by sand fly saliva components has been verified (Lestinova et al., 2017).

Although studies on experimental infection showed initial inocula impacted on clinical course, the parasite burden detected in established lesion may not influence the therapeutic response since Pereira et al. (2017) demonstrated high parasitism in good responders when compared with the poor ones (Pereira et al., 2017). However, there was an increase in parasite burden with recurrent relapses which suggests a failure of the immune system in controlling the parasite replication (Pereira et al., 2017).

Summarizing, several evidence has pointed out the role of parasites in the outcome of the disease. However, as it is not observed in all cases, other factors such as the host immune response and comorbidities, among others, may play a role in both severity and treatment relapse in TL.

## The Host Point of View

### The Impact of Comorbidities on Clinical Manifestations

Many host factors have been associated with the development of skin or mucosal lesions and worsening of TL, as well as the appearance of unusual clinical presentations of cutaneous leishmaniasis. For example, patients infected with *L. major* and affected by diabetes mellitus type I showed more extensive and vegetative lesions (Chiheb et al., 2012). In another study, an association of the nutritional status and clinical and therapeutical evolution in adults and the elderly with ATL was observed (Oliveira et al., 2013). In this study, the impairment in food intake due to the mucosal lesions led to a serum albumin depletion which impaired the healing of the lesions and affected the effectiveness of ATL treatment (Oliveira et al., 2013).

Malnutrition is classified in different forms as follows: (1) undernutrition (wasting, stunting, underweight), (2) inadequate vitamins or minerals, (3) overweight, (4) obesity, and (5) resulting diet-related non-communicable diseases (World Health Organization [WHO], 2017b). WHO estimates that 462 million adults are underweight, 155 million children are stunted and 17 million are severely wasted (World Health Organization [WHO], 2017b) and a considerable part of them is living in endemic areas of leishmaniasis in South America, Africa, and Asia. One among the first studies on the impact of nutritional status on the infection by dermotropic *Leishmania* species was conducted in 1979 using C57BL/6 mice and experimental infection with *L. mexicana* (Pérez et al., 1979). Well-nourished mice presented self-healing lesions, whereas protein malnourished animals had a progressive disease, an impairment of the immune response, characterized by depressed delayed hypersensitivity response and *in vitro* lymphocyte reactivity to *Leishmania* antigen, and failed to recover from *L. mexicana* infection (Pérez et al., 1979). In humans, an association between malnutrition and mucosal lesion development was reported (Machado-Coelho et al., 2005). The impact of malnutrition on the capacity of the immune system to respond to *Leishmania* spp. infection was also observed in a model of murine visceral leishmaniasis (Ibrahim et al., 2013). There, the authors showed that protein malnutrition affects the conduit systems of lymph nodes impacting the barrier function of the organ and consequently promoting parasite dissemination and replication (Ibrahim et al., 2013). In mice, malnutrition results in drastic dysregulation of T cells and cytokine expression and affects the cell-mediated immune response to *L. infantum* by altering T cell migration in the spleen and thymus (Cuervo-Escobar et al., 2014; Losada-Barragán et al., 2017). Although the mechanisms involved in the impairment of the immune response to visceral leishmaniasis in malnourished individuals are better described, they are still unknown for cutaneous leishmaniasis.

In a case-series study conducted in Argentina, 95 patients were evaluated and most of them were diagnosed as infected by *L. braziliensis* (García-Bustos et al., 2016). High prevalence of mucocutaneous cases (35.8%) was detected and most patients with concomitant infectious pathologies, such as Chagas disease, toxoplasmosis, syphilis and others, presented the mucosal form of the disease (62.5%) (García-Bustos et al., 2016). The association between comorbidities and mucosal lesion development was also observed in patients from Brazil (Cunha et al., 2015; Azeredo-Coutinho et al., 2016). Systemic arterial hypertension (58.6%), cardiopathies (mainly cardiac failure) (34.5%) and diabetes mellitus (24.1%) were the most frequent comorbidities observed (Cunha et al., 2015). In addition, the presence of intestinal helminthiasis has been described as able to interfere in both, treatment response and wound closure, as well as the association with mucosal lesions (O’Neal et al., 2007; Azeredo-Coutinho et al., 2016). However, conflicting results have likewise been published showing no influence of intestinal infection in the improvement of clinical outcome (Newlove et al., 2011). Difference in helminthes in genus/species as well as the intensity of infection could explain these differences.

### Does Bacteria and Virus Co-infection Influence the Healing Process in LCL?

Secondary bacterial infections in cutaneous lesions of leishmaniasis are very common and the most frequent bacterium observed is *Staphylococcus aureus* (Ziaie and Sadeghian, 2008; AlSamarai and AlObaidi, 2009; Layegh et al., 2015; García-Bustos et al., 2016; Antonio et al., 2017). In addition, Salgado et al. (2016) have shown diversity in bacteria isolates obtained from lesions and the contralateral healthy skin of the same patient. The knowledge of the impact of secondary bacterial infection on the healing of cutaneous lesions is controversial. Some results do not demonstrate a correlation between bacterial infection and time for wound conclusion in LCL. Layegh et al. (2015) described that the simultaneous treatment for microbial agents did not influence on the healing of cutaneous lesions. Antonio et al. (2017) showed that the presence of secretion and burning sensation produced by bacterial infection influenced epithelization but not the total healing time. On the other hand, several authors have demonstrated the influence of secondary bacterial infections on lesion development and healing delay (Vera et al., 2001; Ziaie and Sadeghian, 2008; Sadeghian et al., 2011; García-Bustos et al., 2016). In this context, the maintenance of neutrophils in active lesions even with more than 4 months duration may be related to the stimulus generated by the persistent amastigotes but also to the presence of bacterial secondary infection (Morgado et al., 2008; Morgado et al., 2015). The inflammatory response to this secondary bacterial infection could hamper self-healing leading to the development of a chronic lesion.

Viruses can also be present in TL lesions. In this context, the impact of viral/*Leishmania* co-infections in pathogenesis was evaluated in a murine model infected with *L. major* and lymphocytic choriomeningitis virus (LCMV) (Crosby et al., 2015). They observed that infection with LCMV led to significantly enhanced disease in *L. major*-infected animals. This increased disease is correlated with an infiltration into the leishmanial lesions of NKG2D^+^ CD8^+^ T cells producing granzyme B, but little IFN-γ (Crosby et al., 2015). The depletion of CD8^+^ T cells after viral clearance, as well as blockade of NKG2D, reversed the increased pathology seen in co-infected mice. The authors demonstrated that even pathogens, known to promote a type 1 response, might exacerbate leishmanial infections (Crosby et al., 2015).

### Cutaneous Leishmaniasis in Extreme Ages

Infants and the elderly share high vulnerability to infections (Vasconcellos et al., 2010; Lang et al., 2011). Although all age groups can be affected by cutaneous leishmaniasis, the most affected ones can vary from each region, from infants less than 9 years old to teenagers (aging 10–19 years) or adults (up to 45 years-old) (Aksoy et al., 2016; Brazil, 2017; Khezzani and Bouchemal, 2017). Therapeutic failure is frequently reported among children affected by cutaneous leishmaniasis (Rubiano et al., 2012; Castro et al., 2017), which could probably be associated with an immature immune system and consequently a failure in controlling the parasite load.

The increase in severity of cutaneous leishmaniasis was also associated with the aging (Machado-Coelho et al., 2005; Vasconcellos et al., 2010; Oliveira et al., 2013; Carvalho et al., 2015). Older patients had two-times more chances to develop mucosal lesions (Machado-Coelho et al., 2005). The reasons why elderly patients are more susceptible to severe manifestations are not fully elucidated. However, an increased number of comorbidities such as cardiomyopathy and diabetes mellitus, as well as the continuous use of medicines to treat them can partially explain it. It is also important to highlight the occurrence of immunological senescence (Weinberger et al., 2008), which reduces the capacity to produce cytokines such as IL-2 and IFN-γ (Cillari et al., 1992) and the accumulation of functional regulatory T cells (CD4^+^FoxP3^+^CTLA-4^+^) which promote chronic infectious disease reactivation (Lages et al., 2008). Elderly persons have a decreased number of naive T cells (CD45RA^+^CD28^+^), which display significantly shorter telomeres and have a restricted TCR repertoire (Pfister et al., 2006). In them, the pool of naïve B and T cells is reduced because the bone marrow and thymus suffer from fat deposition impairing the production of new cells (Chambers and Goodell, 2007). Since CD4^+^T cells are important to control the parasite load in cutaneous leishmaniasis (Bittar et al., 2007; Morgado et al., 2008, 2010; Pereira-Carvalho et al., 2013) this can explain the susceptibility of elderly patients to severe manifestations. Peripheral blood mononuclear cells from elderly patients infected with *L. braziliensis* produced less IFN-γ and more IL-10 than the cells from young subjects (Carvalho et al., 2015). Altogether, these data point to the need to search mucosal or disseminated lesions in elderly patients presenting tegumentary leishmaniasis since they produce lower quantities of IFN-γ and higher quantities of IL-10 than adult patients which may contribute to parasite persistence and *L. braziliensis* infection dissemination. In this context, in a case-series study conducted in Argentina, that included 95 patients diagnosed as infected by *L. braziliensis* (García-Bustos et al., 2016), the authors also detected an association between age and the development of mucosal lesions.

### Gender Differences in Cutaneous Leishmaniasis

In the case-series study above mentioned, (García-Bustos et al., 2016), the authors also detected an association between gender and development of mucosal lesions. They observed a predominance of male and attributed it to their occupational activity since they were rural workers. Although other authors showed higher prevalence of male patients (Machado-Coelho et al., 2005; Giavedoni et al., 2015; Brazil, 2017), the opposite could also be observed (Aksoy et al., 2016). In fact, differences on the immune response profile between male and female animals have been reported (Satoskar et al., 1998; Travi et al., 2002). For example, male hamsters were described as presenting more severe disease and higher intralesional expression of IL-4, IL-10, and TGF-β when compared to female animals (Travi et al., 2002). Furthermore, the Th1 response was more intense in female than male mice (Satoskar et al., 1998). Perhaps sex hormones play a role in the differences observed between genders in cutaneous leishmaniasis as previously observed (Baccan et al., 2011). In this context, in hamsters infected with *L. panamensis*, estrogen was associated to an up-regulation of NOS2 expression and nitric oxide (NO) production (Osorio et al., 2008) that could explain the higher resistance to severe lesions by women when compared to men.

### Cutaneous Leishmaniasis and Pregnancy

Immunological alterations observed during pregnancy affect the course of parasitic infections, such as cutaneous and visceral leishmaniasis (Morgan et al., 2007; Verma et al., 2014; Berger et al., 2017). The worsening of ATL lesions during pregnancy is associated with changes in the innate and adaptive maternal immune response that normalizes postpartum (Conceição-Silva et al., 2013). Pregnant women can develop exuberant and atypical lesions (Guimarães et al., 2009; Conceição-Silva et al., 2013), as well as preterm births and stillbirths (Morgan et al., 2007). Pregnant woman showed an increase in Th2 cells and a reduction in type 1 responsiveness (Conceição-Silva et al., 2013) that is important for healing in leishmaniasis. Although T lymphocytes and macrophages are abundant and similar during pregnancy and postpartum, NOS2 *in situ* expression are more intense postpartum than during pregnancy (Conceição-Silva et al., 2013). The increase of NOS2 expression postpartum is accompanied by an increase of IFN-γ, whereas IL-10 does not show alterations *in situ*. Arginase activity increases during pregnancy and declines at the time of birth to levels like those observed in ATL non-pregnant controls. During pregnancy, a transient modulation of maternal immune responses was observed, characterized by the exacerbation of cutaneous lesions, increased parasite burdens, and reduction of IFN-γ and NOS2, indicating a diminished Th1 response, which was restored postpartum and was associated with the resolution of the wound (Conceição-Silva et al., 2013).

Furthermore, Th1 response against *L. major* in pregnant C57BL/6 mice increased implantation failure and fetal resorptions and was correlated with increased IFN-γ and TNF and reduced IL-10 production by placental cells (Krishnan et al., 1996). In a murine model of experimental infection with *L. mexicana* during pregnancy, resorption and fetal death were observed in a pregnant group (Avila-García et al., 2013). Parasite DNA was found in all placentas evaluated confirming that *Leishmania* is transmitted transplacentally and causes fetal damages in this murine model (Avila-García et al., 2013). These data, in part, could suggest and explain the low number of pregnant women infected with *Leishmania* parasites. Altogether, the data showed that immunomodulation of maternal immune responses during pregnancy has beneficial consequences for the fetus and its development, however, favors *Leishmania* parasite growth and lesion development reflecting in exuberant and atypical lesions.

### Cutaneous Leishmaniasis and Immunosuppression

There are numerous causes of immunosuppression in human patients, for example HIV infection, cancer, immunosuppressive treatments and transplantation. The association between immunosuppression and atypical cutaneous manifestations of leishmaniasis was systematically reviewed in a previous study (Meireles et al., 2017) and described as case reports by several authors (Gontijo et al., 2002; Tuon et al., 2007, 2014; Mortazavi et al., 2014; Souza et al., 2017; among others). In a case of a kidney transplant patient infected with *L. braziliensis*, a concurrent cutaneous, visceral and ocular leishmaniasis was observed (Gontijo et al., 2002). Parasites were isolated from different tissues/sites, such as iliac crest, aqueous humor, and vitreous body suggesting that the immunosuppressive drugs favored the dissemination of *Leishmania*, to different organs including immune-privileged areas (Gontijo et al., 2002). Reactivation of mucosal and cutaneous leishmaniasis was also demonstrated in a renal transplanted patient (Tuon et al., 2014), as well as cutaneous leishmaniasis reactivation years after treatment caused by systemic corticosteroids (Tuon et al., 2007).

More recently, cases of patients with severe autoimmune diseases who had to be under therapy involving immunosuppressant drugs such as methotrexate (MTX), monoclonal antibodies (mAbs), mainly TNF-inhibitory mAbs, and prednisone, and who presented tegumentary and/or visceral lesions have been reported even in the absence of a prior compatible history. The association of anti-TNF therapy and the increased risk for developing opportunistic infections has also been associated (Neumayr et al., 2013; Calabrò et al., 2016). Regardless of the fact that most of the cases published showed a reactivation of visceral leishmaniasis, cases of association between rheumatoid arthritis (RA) and ATL were also described (Souza et al., 2017). Despite the increasing amount of information available, most physicians still have problems to rapidly diagnose cutaneous leishmaniasis (CL) or mucosal leishmaniasis (ML) in patients submitted to immunosuppressive treatment since these patients very often show epidemiological evidence of previous leishmaniasis, however, they live in non-endemic regions of neglected tropical diseases at the time of diagnosis.

The worsening of clinical manifestations, multiple lesions and high degree of relapses post-treatment in ATL patients co-infected with HIV were previously described (Rosatelli et al., 1998; Puig and Pradinaud, 2003; Lindoso et al., 2009, 2016; Giavedoni et al., 2015; Calvopina et al., 2017). In a study of fifteen cases of AIDS/TL, Lindoso et al. (2009) observed mucosal lesions progress in 80% of the patients, disseminated lesions in 60% and genital lesions in 27% of the patients. All of them were treated and 56% of the patients showed relapses. The immunological bases of increased immunosuppression of HIV/leishmaniasis co-infected patients have been studied. *Leishmania* infection can increase the degree of immune system activation in individuals concomitantly infected with HIV (Santos-Oliveira et al., 2010). Although the alterations were more intense for VL/HIV-AIDS patients than for TL/HIV-AIDS ones, the authors observed lower CD4^+^ T cell counts and higher proportion of activated T lymphocytes in co-infected patients even when HIV viral load was suppressed under HAART (Santos-Oliveira et al., 2010). In HIV-AIDS patients the decrease in the pool of CD4^+^ T cells and consequent diminution of the CD4/CD8 ratio, produced by HIV infection provokes a generalized immune depression (Da-Cruz et al., 1992). In this study, the patient’s disseminated clinical manifestation was probably related to the inability of the T cell-mediated immune responses to control the spread of *Leishmania* infection (Da-Cruz et al., 1992). HIV/*Leishmania* co-infected patients also present a reduction in the lymphoproliferative response to *Leishmania* antigens associated with the decreased quantity of both effector memory and central memory CD4^+^ T-cells (Góis et al., 2014). A reduction in the IFN-γ and IL-13 levels and the ratio of IFN-γ/IL-10 produced in response to stimulation with soluble *Leishmania* antigens were also observed in individuals infected with HIV and/or cutaneous leishmaniasis (Rodrigues et al., 2011). They suggested that alterations in cytokines expression create a microenvironment that favors the replication and the spread of the *Leishmania* parasites, leading to the dissemination of cutaneous infection and the visceralization of typically dermotropic species (Rodrigues et al., 2011).

## Parasite Persistence

The reactivation of leishmaniasis lesions after clinical cure in immunosuppressed patients led to the discussion on parasitological cure and parasite persistence in leishmaniasis. How and why some parasites persist for long periods, perhaps the entire life of the infected individual? What would be the advantage and the disadvantage of this fact? Many answers have been obtained, but some questions have not been made clear yet.

The inflammatory process is a complex event that produces energy expenditure for its organization, and control, very important step because unbalanced inflammation can produce as much or more tissue damage as the stimulus, which originated it. Therefore, our organism uses several mechanisms to have efficient, precise and fast action. Thus, the inflammation necessary to control an infection may not be sufficient to produce the complete elimination of the infectious agent. In addition, the presence of the pathogen in a residual and controlled manner may even be beneficial in the sense of constantly and limitedly stimulating the immune system; consequently the organism would always be prepared for new infections. The problem is when this interaction parasite-host becomes unbalanced. At this time, the infection may reappear, either by an increase in the virulence of the parasite or by alteration of the immune response that controls the infection.

One of the first pieces of information about the residual presence of *Leishmania* parasites in tissue after the clinical healing was obtained in a murine model. Aebischer et al. (1993) obtained confirmation of the presence of *L. major* in viscera of C57BL/6 mice cured for more than 1 year. In addition, the authors were able to demonstrate the infectivity of the isolated parasites. Parasite persistence has been also demonstrated in clinical cured patients (Schubach et al., 1998; Coutinho et al., 2002; Mendonça et al., 2004; Oliveira-Camera et al., 2006; Morgado et al., 2010; Castro et al., 2017; Martínez-Valencia et al., 2017). *Leishmania* persists in skin and mucosal tissues in a high proportion of patients who achieved therapeutic cure of CL (Martínez-Valencia et al., 2017). The possibility of parasite persistence after clinical cure suggests that the immune response can control, but not fully eliminate the infection (Coutinho et al., 2002; Morgado et al., 2010; Conceição-Silva et al., 2010). In this context, healed leishmaniasis cutaneous lesions were evaluated after 1 and 3-year post-clinical cure (Morgado et al., 2010). Although the patients show the epithelization of lesions, microscopically the amastigotes persist in the skin, as well as the inflammatory reaction as focal or nested infiltration. The equilibrium between parasite and host is progressively achieved over the years with the reduction of inflammatory cells at the site of lesion (Morgado et al., 2010). Once achieved, this equilibrium is maintained by the relation between T reg cells and effector lymphocytes (Mendez et al., 2004). Trauma, elderly patients, comorbidities, co-infections and immunosuppression can destabilize this equilibrium leading to reactivation of the lesion (Oliveira-Neto et al., 1998; Lages et al., 2008; Azeredo-Coutinho et al., 2016; Souza et al., 2017; Martínez et al., 2018).

In the murine model experimentally infected with *L. major*, parasite persistence in the skin is mediated by CD4^+^CD25^+^ Treg cell accumulation (Belkaid et al., 2002; Yurchenko et al., 2006). CD4^+^CD25^+^ nTreg cells migrate to *L. major*-infected dermal sites through the CCR5 signaling, promoting the establishment of infection and long-term survival of the parasite in the host (Mendez et al., 2004; Yurchenko et al., 2006). In the lesion site, CD4^+^CD25^+^ T cells suppress the ability of CD4^+^CD25^-^ effector T cells to eliminate the parasite by both, IL-10-dependent and IL-10-independent mechanisms (Belkaid et al., 2002; Viana da Costa et al., 2002). The effector and regulatory T cells balance established in sites of chronic infection might reflect both parasite and host survival strategies since the sterilizing immunity achieved in mice with impaired IL-10 activity is followed by the loss of immunity to reinfection (Belkaid et al., 2001, 2002). Fibroblasts were identified as safe cell targets for the *Leishmania* parasites in clinically latent disease (Bogdan and Röllinghoff, 1998). When compared with macrophages, these cells showed a reduced ability to express type 2 nitric oxide synthase and to kill intracellular *L. major* (Bogdan and Röllinghoff, 1998). Stenger et al. (1996) showed the reactivation of latent leishmaniasis by inhibition of inducible nitric oxide synthase in mice experimentally infected with *L. major*. However, recently two populations: one rapidly replicating, like parasites in acute infections, and another showing little evidence of replication were demonstrated in the persistent infection by *L. major* (Mandell and Beverley, 2016, 2017). The persistent parasite were found residing in macrophages and DCs expressing inducible nitric oxide synthase (iNOS), instead of “safe” immunoprivileged cell types, suggesting that some populations of *Leishmania* may be resistant to NO (Mandell and Beverley, 2017). *L. major*-infected dendritic cells and macrophages in lymph nodes of immune animals representing long-term host cells were demonstrated a long time ago (Moll et al., 1995). Once dendritic cells could present endogenous parasite antigen to T cells, long-term infected dendritic cells may thus allow the sustained stimulation of a population of parasite-specific T cells, protecting the mice from reinfection favoring the maintenance of T cell memory (Moll et al., 1995).

There are various mechanisms of immune escape described for *Leishmania*, which led to parasite persistence in the host. They include: passive protection of the parasite against antileishmanial products and retreat into “safe target cells,” active suppression of the synthesis of reactive oxygen or nitrogen intermediates, modulation of the host cytokine response, inhibition of antigen-presentation and T cell-stimulation, and induction and expansion of counterprotective Th cells (Bogdan and Röllinghoff, 1998). Despite the fact that these mechanisms alone are not able to guarantee the survival of the parasite, together they might provide the safe environment protecting the parasite from elimination (Bogdan and Röllinghoff, 1998).

## Concluding Remarks

**FIGURE 3:**
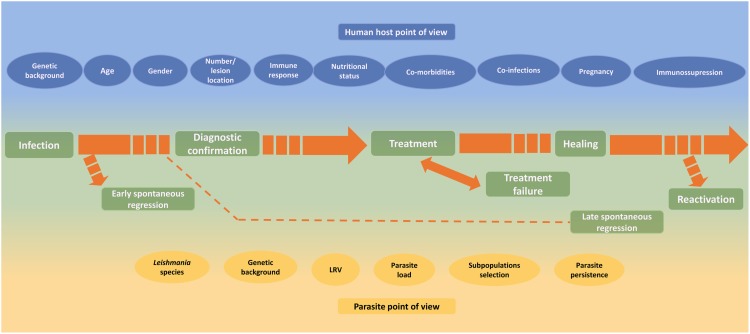
Host and parasite factors that may influence the course of infection, the lesion healing and the reactivation of cutaneous leishmaniasis.

Different factors such as *Leishmania* species, host genetic background, age, nutritional status, comorbidities, lesion duration, number and localization of lesions, drug metabolism, irregular treatment, individual host cellular immune response, among others were described as capable of influencing the TL outcome (**Figure 3**). Unfortunately, despite an impressive amount of information, a conclusive understanding about the magnitude of their roles remains under construction. In addition, a multifactorial influence cannot be discarded. A better knowledge of the role of different factors from either parasites or patient sites can improve the understanding of the influence of these factors in the healing process, clinical healing maintenance and reactivation of human tegumentary leishmaniasis. This knowledge is fundamental to better understand disease progression, thus allowing better treatment and control, and improvement in patient care.

## Author Contributions

FC-S, JL-S, and FM conceived the manuscript. FC-S and FM conceived the figures. FM prepared the figures. JL-S prepared the tables. FC-S, JL-S, and FM wrote, reviewed, and approved the manuscript.

## Conflict of Interest Statement

The authors declare that the research was conducted in the absence of any commercial or financial relationships that could be construed as a potential conflict of interest.
